# Effects of *Salvadora persica* Extract on the Hematological and Biochemical Alterations against Immobilization-Induced Rats

**DOI:** 10.1155/2015/253195

**Published:** 2015-06-28

**Authors:** Kholoud S. Ramadan, Salha A. Alshamrani

**Affiliations:** Department of Biochemistry, Faculty of Girls Science, King Abdulaziz University, Jeddah, Saudi Arabia

## Abstract

A total of 24 rats were divided into 4 groups: control, stress, extract alone, and stress + extract (*n* = 6 each), for total 21 days of treatment. The immobilization stress was induced in rats by putting them in 20 cm × 7 cm plastic tubes for 2 h/day for 21 days. Rats were postorally treated with* Salvadora persica* at a dose of 900 mg/kg body weight via intragastric intubations. At the end of the test period, hematological and biochemical parameters were determined in blood and serum samples with determination of vital organs weights. The vital organ weights were not significantly affected in stressed rats as compared to control rats. Compared to the control group, the stress treated group showed significances in several hematological parameters, including decreases in WBC, RBC, and PLT counts. Furthermore, in comparison to the control group, the stress group showed significantly increased blood glucose, serum total cholesterol, LDL-cholesterol, and triacylglycerols levels and decreased HDL-cholesterol level. The hematological and biochemical parameters in the stress + extract treated group were approximately similar to control group. The SP extract restored the changes observed following stress treatment.

## 1. Introduction

Stress can be considered as an important main cause of many diseases like coronary artery diseases, cancer, and diabetes mellitus [1]. Stress has become an increasingly popular and widely applied term in our everyday languages. People have witnessed the physical damage of stress that can work on the body [2].

Immobilization is a suitable and easy method to cause physical stress which leads to restricting mobility and aggression in animal model [3]. However, raising evidence has implied that the production of free radicals plays an important role in this process.

Focusing on the usage of natural antioxidants is a new strategy for mitigating oxidative damage. Many of the negative effects of oxidative stress are decreased after supplementation with certain dietary antioxidants [4]. There is an increasing interest in total medicinal plant extracts, the largest value of which may be due to their constituents that subscribe to the modulation of the oxidative balance* in vivo*. Additionally, the special advantage of total plant extracts is that they are easily accessible products, without purification, to apply them in possible prevention of diseases [5].

The medicinally important species of* Salvadora persica* L. (SP), also known as Miswak, mustard tree, and toothbrush tree, distributed mainly in tropical and subtropical Asia. Miswak belongs to the family of Salvadoraceae and every part of the plant is used as a medicinal purpose among global Muslim community. Various phytochemical studies on* Salvadora persica* reported the presence of alkaloids, salvadorine, flavonoids, steroids, trimethylaine, and salvadoricine. Various ingredients of* Salvadora persica* have valuable important biological properties, including critical antibacterial, antifungal activity and antidiabetic [6, 7].

Many studies reported that oral ingestion of medicinal drugs can alter the hematological parameters ranges to either positive or negative [8]. Many of these therapeutic effects have been confirmed by contemporary scientific research and their antistress effects have not been well researched. Therefore, this study was planned to investigate the antistress effects of administration of* Salvadora persica* extract on hematological and biochemical alterations induced by immobilization stress in rats.

## 2. Materials and Methods

### 2.1. Collection and Authentication of Plant

*Salvadora persica* L. (SP) was purchased from local market in Jeddah, Kingdom of Saudi Arabia, and authenticated by Herbarium, King Abdulaziz University.

### 2.2. Preparation of Aqueous Extract

The fresh Miswak root sticks were cut into small pieces and allowed to dry at room temperature for one week. Then they were ground in grinding machine to fine powder, mixed with distilled water, and extracted for 24 h at 150 rpm at 25°C in a shaker. The mixture was then centrifuged at 3000 rpm for 20 min. The supernatants were subsequently filtered through Whatman No. 1 filter paper and the filtrate was concentrated in rotary evaporator (Buchi Rotavapor R-200) at 70°C and was lyophilized. The resulting powder was packed in a glass bottle and stored at 4°C until needed. It was dissolved in distilled water to prepare the exact aqueous dose (900 mg Kg^−1^ body weight) for intragastrical injection [9].

### 2.3. Animals and Experimental Design

Adult male albino rats (weighing 120–160 g) were obtained from Central Animal House in Jeddah, Saudi Arabia. The animals were housed in acrylic cages in standard conditions of temperature before starting the experiments to adapt to the laboratory condition and fed with commercial diet and water ad libitum. The experimental protocol was approved by the Institutional Animal Ethical Committee (IAEC) of Saudi Arabia, Jeddah.

The immobilization stress was induced in rats by putting them in 20 cm × 7 cm plastic tubes for 2 h/day for 21 days [10, 11]. There are several 3 mm holes at the far end of the tubes for breathing that allows ample air but animals will be unable to move. Twenty-four rats were randomly divided into four groups of six animals each. Experimental groups were designed as follows: control group that received distilled water; stressed group;* Salvadora persica* (SP) that received daily extract (900 mg/Kg bw) intragastrically using animal feeding intubation needles at the same time of day, for 21 days; stress + extract group. Rats were subjected to 2 h stress once a day for a period of 21 days except the nonstress group. Following stress session, rats were returned to home cages and were able to access food and water freely for the remainder of the day. Rats in different groups were weighed on days 0, 7, 14, and 21. They were closely observed for behavioral and general morphological changes.

### 2.4. Vital Organs Weights

At the termination of treatment, 21 days, vital organs (heart, brain, liver, and kidneys) were harvested from scarified rats. They were washed with ice-cold saline solution (0.9% w/v), blotted, and weighted. The weight of each organ was standardized to 100 g body weight of each animal.

### 2.5. Hematological Assay

Blood samples were collected from rats into heparinized tubes under light ether anesthesia. White blood cells (WBC), lymphocyte and monocyte ratio, red blood cells (RBC), hematocrit (Hct), hemoglobin (Hb), mean cell volume (MCV), mean cell hemoglobin (MCH), mean cell hemoglobin concentration (MCHC), and platelet count (PLT) were measured on Hematology Analyzer (Abacus Junior Vet 5, Austria).

### 2.6. Biochemical Analysis

At the end of the experimental period, animals were fasted for 8–12 h before blood collection in order to cause no interference in the analysis of blood glucose and serum lipid profile. Blood samples were withdrawn by end tail vein cutting method from overnight fasted animals and blood glucose was measured by one touch electronic glucometer ACU check. Other blood samples were collected and allowed to coagulate at room temperature for 30 min and were subsequently centrifuged at 3000 g for 10 min. Serum was removed and stored at −80°C until analysis.

Estimation of biochemical parameters such as serum total cholesterol [12], triacylglycerols [13], and HDL-cholesterol [14] was measured. The LDL and VLDL levels were calculated using the formulae [15].

### 2.7. Statistical Analysis

Data were analyzed by one-way analysis of variance (ANOVA) followed by Student's *t*-tests using a commercially available statistics software package (SPSS for Windows, V. 15.0) program. Results were presented as means ± SD. *p* values <0.05 were regarded as statistically significant.

## 3. Results

### 3.1. Effect of SP on Body Weight

**Figure 1:**
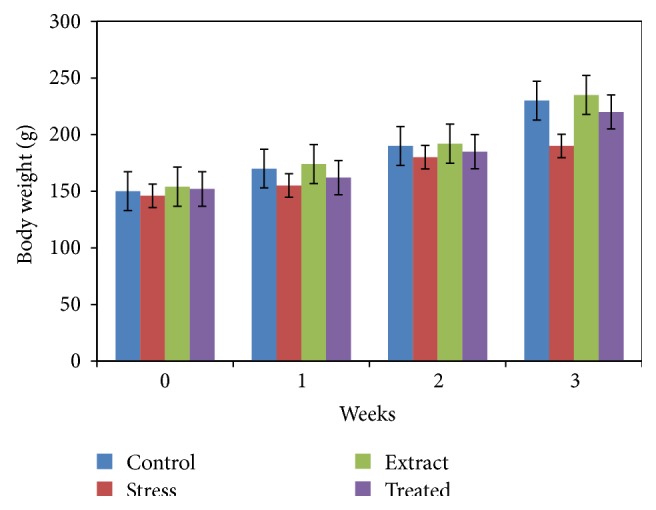
Effects of SP extract (900 mg/kg bw) on body weight in control and immobilization-induced stress rats (21 days). The results are expressed as the mean ± SD for 6 animals in each group.

The effects of SP pretreatment on body weight are shown in Figure 1. The body weight change is a physical parameter that associates with the stress response. As shown in Figure 1, rats in the nonstressed control group had a normal weight accumulation, whereas the weight gain in the immobilization stress group was significantly lower than the control group. However, the administration of SP extract significantly slowed the immobilization stress-induced body weight loss. As a result, at 21 days, the rats in the immobilization stress group lost on average 19% of their maximal body weight, whereas the body weight of the rats treated with SP extract with immobilization stress was reduced by 8%. Thus, treatment with the SP significantly reversed the stress-induced weight loss.

### 3.2. Effect of SP on Vital Organs

The SP extract did not produce any significant effect on the weight of various vital organs of rats after daily administration for 21 days. Minor significant increase in brain weight of stress rats was observed (Table 1).

### 3.3. Effect of SP on Hematological Parameters in Control and Immobilization-Induced Stress in Rats

The SP treated rats exhibited significant (*p* < 0.01) improvement in most of the hematological parameters and red blood cells indices compared to stress rats (Table 2). The data showed that immobilization stress caused a significant decrease in WBC, RBC, and PLT counts as compared to control group. The hematological parameters in stress + extract treated group were similar to control group. The SP extract alleviated the adverse effects on WBC, RBC, and PLT counts caused by stress.

### 3.4. Effect of SP on Biological Parameters in Control and Immobilization-Induced Stress in Rats

**Figure 2:**
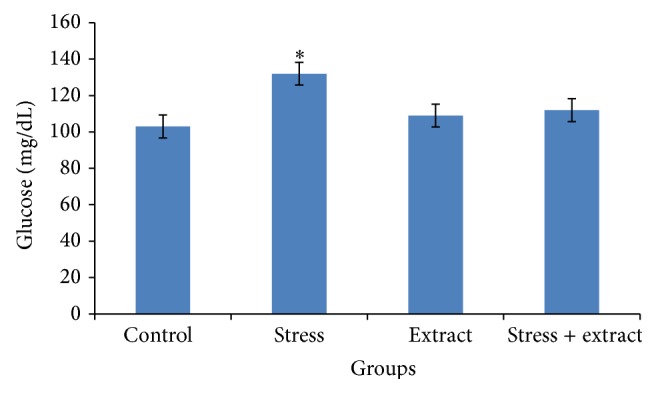
Effects of SP extract (900 mg/kg bw) on blood glucose level in control and immobilization-induced stress rats (21 days). The results are expressed as the mean ± SD for 6 animals in each group.

Exposure to immobilization stress resulted in an increased blood glucose level in stressed rats (Figure 2), which was significantly decreased by SP extract at a dose of 900 mg/kg when compared to stress rats.

The effects of SP treatment on serum lipids profile of control and stress animals are listed in Table 3. Immobilization stress elevated serum cholesterol, triacylglycerols, and LDL-cholesterol and decreased HDL-cholesterol levels in stressed rats. SP extract at a dose of 900 mg/kg significantly decreased the higher levels of cholesterol, triacylglycerols, and LDL-cholesterol and significantly increased the HDL-cholesterol levels compared to stress rats (Table 3).

## 4. Discussion

The immobilization stress can be considered the most severe type of stress in animal models and has a comparative effect on humans. In the present study, rats in the control group had a normal weight accumulation, whereas the weight gain in the immobilization stress group was significantly lower than the control group. The administration of* Salvadora persica* (SP) extract significantly slowed the immobilization stress-induced body weight loss. The decrease in body weight may be due to the decreased food intake in the rats under the influence of stress. Besides that the decrease in the body weight might also have associated with stress-induced increase in metabolic demands, reduced digestion, and increased adrenal steroid secretion [3].

Stress is thought to impair immune function [16, 17] through emotional or behavioral manifestations such as anxiety, fear, tension, cognition, anger, and sadness and physiological changes of heart rate, blood pressure, glucose metabolism, and sweating.

The present study indicated that stress-induced significant adverse effect on hematological parameters in rats 21 days after treatment with SP had successfully ameliorated the hematological disturbances induced by stress. The effect of stress was demonstrated by the significant reduction in WBC, RBC, and PLT counts. Similar to our studies, others have shown decreases in WBC and RBC counts in animals exposed to immobilization stress [18]. Some studies reported that immobilization changes in blood parameters such as Hb, HCT, MCV, MCH, and MCHC [19], but in this study no statistical differences were observed in these parameters. The increase in the hematological constituents in the stress loaded group cotreated with SP might be due to the antioxidant, antilipid peroxidation, and anticonvulsant effects of SP.

Increased levels of glucose are seen in the immobilization stressed rats due to the decreased secretion of insulin level [20]. In response to stress, adrenocorticotropic hormone (ACTH) is released which acts on adrenal cortex which by cortisol and corticosterone will be secreted. Treatment with SP significantly decreased blood glucose when compared to stress rats [21].

The regular raised blood glucose like in condition of chronic stress depressed the cognitive functions and immune function. In this study the SP extract (900 mg/kg) showed regulatory effect on circulating glucose significantly in stress conditions. The antidiabetic activity of SP may be due to the presence of phytochemicals (flavonoids, tannins, glycosides, sterols, and saponins) [22]. Plants that contain the active principals such as glycosides and flavonoids have antioxidant activity and are said to possess antidiabetic effect. Besides this, SP also contain several organic sulphur compounds and it is well known that sulphur derivatives show hypoglycemic effects. In fact, many plants containing sulphur are used traditionally as antidiabetic [23].

Hyperglycemia is associated with dyslipidemia and is a risk factor for coronary heart diseases. The higher lipid profile of stressed rats was due to increased mobilization of free fatty acids from peripheral depots and also due to lipolysis caused by hormones. Under normal circumstances insulin activates the enzyme lipoprotein lipase which hydrolyses triacylglycerols. The dyslipidemia is characterized by increase in total cholesterol and triacylglycerols and decrease in HDL-cholesterol. This abnormal serum lipid profile was corrected towards normal after treatment with aqueous extract of SP. The level of cholesterol was significantly higher in stress group which is similar to those reported by other researchers [24]. The increased level of triacylglycerol may be also due to the stress-induced catecholamine surge [3]. A possible mechanism of aqueous extract may be due to the presence of flavonoids, which significantly increase LDL receptor mRNA levels, which, in turn, increase hepatic uptake and degradation of LDL causing a decrease in serum LDL levels [25]. Hence it can be concluded that SP extract has revealed significant effect owing to its ability to reduce level of blood glucose, total cholesterol, and triglyceride and increase HDL level [26].

## 5. Conclusion

The study therefore concluded that the aqueous extract of* Salvadora persica* (SP) has potential antistress activity. SP prevented the stress-induced abnormalities in hematological parameters, glucose, and lipid profile indicating the protective effect against stress. Further experimentation needs to be done with regard to fraction of extract, isolation, and characterization to explore active constituents responsible for such activity and to elucidate the possible biochemical mechanism.

## Figures and Tables

**Table 1:** Effects of SP extract on rat organs weights (per 100 g body weight) in control and immobilization-induced stress rats.

Groups	Relative organ weight			
	Liver	Kidney	Brain	Heart
Control	3.43 ± 0.2	0.76 ± 0.06	0.69 ± 0.03^a^	0.3 ± 0.03
Stress	3.45 ± 0.5	0.77 ± 0.07	0.8 ± 0.08^b^	0.32 ± 0.04
SP extract	3.46 ± 0.32	0.78 ± 0.1	0.69 ± 0.02^a^	0.32 ± 0.001
Stress + extract	3.33 ± 0.4	0.62 ± 0.2	0.73 ± 0.04^a^	0.33 ± 0.03

All the values are expressed as mean ± SD (*n* = 6 for each group). Values not sharing a common alphabet as superscripts are significantly different from each other at the level of *p* < 0.05.

**Table 2:** Hematological parameters in control and experimental groups.

	Control	Stress	SP extract	Stress + extract
WBC (10^3^/mm^3^)	8.48 ± 0.3^a^	5.2 ± 0.03^b^	11.2 ± 0.36^c^	10.3 ± 0.8^c^
Lymphocyte (%)	85.33 ± 2.8	90.6 ± 2.2	89.9 ± 3.1	89.8 ± 3.2
Monocytes (%)	11.2 ± 2.6	7.23 ± 0.4	9.45 ± 2.2	7.73 ± 1.5
RBC (10^6^/mm^3^)	7.33 ± 0.35^a^	6.8 ± 0.25^b^	7.6 ± 0.36^a^	7.9 ± 0.46^a^
HGB (g/dL)	13.6 ± 1.3	11.7 ± 1.7	13.8 ± 0.6	12.7 ± 0.8
HCT (%)	41.2 ± 1.2	34.6 ± 2.3	40 ± 2.6	46.3 ± 0.9
MCV (fL)	56.06 ± 0.5	57.56 ± 1.7	57.4 ± 0.004	58.5 ± 1.9
MCH (pg)	18.38 ± 0.49	17.75 ± 0.5	18.15 ± 0.19	17.5 ± 0.57
MCHC (g/dL)	32.4 ± 0.2	31.07 ± 0.3	31.83 ± 0.02	31.4 ± 0.1
PLT (10^3^/mm^3^)	809 ± 38^ac^	676.7 ± 79^b^	1071.3 ± 37^a^	747 ± 87^c^

All the values are expressed as mean ± SD (*n* = 6 for each group). Values not sharing a common alphabet as superscripts are significantly different from each other at the level of *p* < 0.05.

**Table 3:** Effect of SP extract on lipid profile in experimental animals.

Groups	Control	Stress	SP extract	Stress + extract
Cholesterol (mg/dL)	40.4 ± 1.5^a^	58.8 ± 1.7^b^	45.1 ± 2^a^	44.5 ± 2^a^
HDL-c (mg/dL)	37.08 ± 1.9^a^	33 ± 3.7^b^	36.8 ± 1.9^a^	38.78 ± 4.4^a^
Triacylglycerol (mg/dL)	65.3 ± 5^a^	103.8 ± 22^b^	65.7 ± 10^a^	83.1 ± 14^a^
LDL-c (mg/dL)	7.33 ± 0.85^a^	25.2 ± 0.9^b^	8.1 ± 1^a^	9.1 ± 1.46^a^

All the values are expressed as mean ± SD (*n* = 6 for each group). Values not sharing a common alphabet as superscripts are significantly different from each other at the level of *p* < 0.05.

## Abbreviations

SP: * Salvadora persica*
RBC: Red blood cells
WBC: White blood cells
PLT: Platelets.
